# Books Reviewed

**Published:** 1868-11

**Authors:** 


### Books Reviewed.

Vesico-Vaginal Fistula. By Thomas Addis Emmet, M. D. New York: Wm. Wood & Co., 1868.

The author in his introductory remarks says, that "prior to the application of the metallic suture by J. Marion Sims, and a proper mode of exploration furnished by his speculum, any attempt for the relief of the injuries under consideration was uncertain in result, and the operation regarded as an opprobrium to the profession. Since that time, however, it has become more certain of success than any other operation in surgery, and long since should have ceased to be confined to the hands of comparatively a few operators. In truth, no more brains or tact is needed in the execution of this than in many other operations of surgery which have long since become familiar to the many." We admire the fairness and absence of pretension which is embodied in these declarations, and believe the author has done himself much credit in his open declaration that all surgeons can operate successfully for vesico-vaginal fistula. It is not to be supposed that all surgeons can make this operation with the same ease and adroitness with which Dr. Emmet can make it, who has already perfected himself by several hundred operations, but all surgeons have Dr. Emmet's assurance that they can do it successfully with the same ease and certainty with which other operations in surgery are made by them. For this assurance, all who propose to make the attempt will be very much obliged.

It is undoubtedly true, that prior to the applicaiion of the metallic suture by Dr. Sims, and the mode of exploration furnished by his speculum, operations for vesico-vaginal fistula were uncertain in result, and were not looked upon so favorably or attempted so frequently as since. Dr. Sims may be said to have called the attention of the profession to the feasibility and safety of this operation, and to have done it in connection with his announcement of the application of the metallic suture. Metallic sutures, however, and Sim's speculum are neither of them indispensable in successfully making operation for vesico-vaginal fistula. The announcement of the use of the silver wire in place of silk, was not always made in good taste, or the modes of drawing and securing these sutures as described by him, either convenient or necessary, yet it had the effect of drawing attention to the relief of this and kindred maladies, and it is all right, when fairly understood, to date the popularity of this operation from that time.

The chief object of the author appears to have been to present the profession a collected record of the most interesting and remarkable cases furnished him by his extensive experience in this form of disease. We are at first astonished at the number of extraordinary and remarkable cases he reports, but a moment's reflection explains the mystery. The author's connection with the Woman's Hospital, almost since its foundation, has given him an unexampled experience, since this hospital was founded mainly for the relief of this and similar affections, and has attracted patients from all portions of the country by the liberality of its provisions. We have a detailed account of seventy-five cases, illustrating nearly every condition this malady can possibly assume, and furnishing an invaluable guide in the management of similar cases. Many cases were so severe and fearful in nature and extent, that outside this book no encouragement to attempt interference could be found; but if disorganization so extensive can be repaired, there can hardly be conceived a condition of destruction of these parts, so fearful as not to leave hopes of repair. The work is written in a most instructive and unpretending style, and conveys to the reader a clear understanding of the various subjects discussed. The author is entitled to the hearty thanks of the profession for his liberality in thus offering the results of his experience for the guidance of all who may desire to avail themselves of an observation based upon a greater number of facts than could perhaps be presented by any other physician of this country.

The work is illustrated where necessary to convey correct idea of the text; is published in appropriate style by William Wood & Co., of New York, and will be most attentively and profitably read by all surgeons who attempt the treat_ ment of vesico-vaginal fistula and many other similar maladies.

---

Transactions of the Medical Society of the State of North Carolina, 1868.

The first Paper which attracts our attention, is the Address by Dr. A. B. Norcom, from which we have made a short extract, we regret we are unable to publish it complete; it is worth careful perusal by every practicing physician.

The second Paper is the retiring President's Address, which is also a very creditable production. The only strictly medical paper is a report of a case of gun-shot wound healed by first intention.

---

Transactions of the Indiana State Medical Society.

In this volume is published the Prize Essay of James R. West, M. D., of Richmond, Indiana, upon the causes, nature and treatment of Cerebro-Spinal Meningitis. A report on Cholera, by A. Field, M. D., of Jeffersonville, Ind. Reports upon "Indiana's Idiotic Children," by H. P. Ayers, M. D., of Fort Wayne, Ind. Placenta Previa, by G. W. Mears, M. D., of Indianapolis, Ind. Diseases of Women, by Theophilus Parvin, M. D., of Indianapolis, Ind. Various reports of interesting and remarkable cases, and a report on Cholera, by James F. Hibberd, M. D., Richmond, Ind. The transactions of the Society, and the President's Address, are also comprised in the volume. Many of the Papers are highly meritorious, and all are creditable.

---

Atlas of Venereal Diseases. By A. Cullerier. Translated from the French by Freeman J. Bumstead, M. D. Part V. Philadelphia: Henry C. Lea; 1868.

This completes the volume, which contains one hundred and fifty beautifully colored figures, which illustrate the whole subject most admirably. Dr. Bumstead has placed the American Medical Profession under lasting obligations by this translation, with his notes and additions. This work upon venereal diseases must rank with the first in the English language, and cannot be excelled in the truthfulness and beauty of its illustrations and in the accuracy and correctness of its teaching. The student must prefer it to all others, since it combines the most modern views of the nature of these diseases, with life-like representations of all their varied appearances, and the general practitioner will give it preference, since at a glance the whole subject is fully open before him. It is a very valuable book, and all who desire to know about the diseases in question should not fail or delay in obtaining it. For the numbers as supplied from time to time, we are also furnished with an appropriate cover, in which the parts can easily be placed by the binder, and when thus complete the volume is one of real value and beauty.

---

Criminal Abortion: Its Nature; its Evidence, and its Law. By Horatio R. Storer, M. D. Franklin and Fisk, Heard. Boston: Little, Brown & Co; 1868.

All our readers are familiar with the previous works of Prof. Storer, upon the subject of criminal abortion, and understand how much he has done to impress both the profession and public with its frequency; its evil effects upon the mother; its influence upon the race; and above all, its crime. Whatever objection may be made to the manner in which his publishers have advertised his work, or the means deemed necessary to introduce it to the public, it must not be denied but the subject has been fearlessly, faithfully, and ably discussed, and that one of the most barbarous crimes, long practiced and concealed by a false and mistaken modesty, has been faithfully and publicly exposed. The present volume contains the re-written arguments presented in previous works by the same author, and these have been joined with the legal questions involved in the prosecution of this crime; the common law; the English statutes and indictments thereon; against whom an indictment lies; principals and accessories; indictment; evidence, etc.; making a volume of over two hundred pages, which must be highly acceptable to the professions of both law and medicine. Much labor has been bestowed in making the legal bearings of this crime complete and accurate. In writing that portion which treats of the criminal law, it has been the object of the authors to exhaust the subject, so that the work may be regarded, in this respect, complete for guidance both in prosecution and defense.

These arguments require to be re-written and re-published and re-read. The mass of the public are yet wholly ignorant of the crime—of its nature, its effects, and its sin. The professiens are not yet fully aware of the bearings of the various questions involved, and we know of no way in which they can be better enlightened than by perusal of this, and the previous writings of Prof. Storer. After all that may be known, we fear it will prove of like history with prostitution: no law, and no penalty, sufficient to perceptably diminish the crime.

---

Ovariotomy: A Paper read before the Ohio State Medical Society. By Alexander Dunlap, M. D.

The author gives his experience in forty operations for ovariotomy, nine of which, only, were fatal. In all instances the pedicle was secured by double ligature; the ends being drawn through the lower angle of the wound—drawn tightly, but the pedicle not placed upon the stretch. The tumors weighed from fourteen to one hundred and thirty-six pounds; two were solid, and one of these was fibrous and bony, weighing one hundred and six pounds; patient died the eleventh day. The other weighed thirty-two pounds; the patient died from hemorrhage. The author has had an extensive and interesting experience, and his Paper is an original and instructive one.

